# Disc repositioning by open suturing vs. mini-screw anchor: stability analysis when combined with orthognathic surgery for hypoplastic condyles

**DOI:** 10.1186/s12891-022-05337-2

**Published:** 2022-04-26

**Authors:** Jiangshan Hua, Chuan Lu, Jieyun Zhao, Zhi Yang, Dongmei He

**Affiliations:** 1 Department of Oral SurgeryShanghai Key Laboratory of Stomatology &National Clinical Research Center of Stomatology, Ninth People’s Hospital, Shanghai Jiao Tong University School of Medicine, Shanghai Research Institute of Stomatology, Shanghai, China; 2 Department of Oral and Cranio-Maxillofacial SurgeryShanghai Key Laboratory of Stomatology &National Clinical Research Center of Stomatology, Ninth People’s Hospital, Shanghai Jiao Tong University School of Medicine, Shanghai Research Institute of Stomatology, Shanghai, China

**Keywords:** Temporomandibular joint, Anterior disc displacement, Jaw deformity, Disc repositioning, Orthognathic surgery

## Abstract

**Background:**

Disc repositioning by Mitek anchors for anterior disc displacement (ADD) combined with orthognathic surgery gained more stable results than when disc repositioning was not performed. But for hypoplastic condyles, the implantation of Mitek anchors may cause condylar resorption. A new disc repositioning technique that sutures the disc to the posterior articular capsule through open incision avoids the implantation of the metal equipment, but the stability when combined with orthognathic surgery is unknown. The purpose of this study was to evaluate the stability of temporomandibular joint (TMJ) disc repositioning by open suturing in patients with hypoplastic condyles when combined with orthographic surgery.

**Methods:**

Patients with ADD and jaw deformity from 2017 to 2021 were included. Disc repositioning by either open suturing or mini-screw anchor were performed simultaneously with orthognathic surgery. MRI and CT images before and after operation and at least 6 months follow-ups were taken to evaluate and compare the TMJ disc and jaw stability. ProPlan CMF 1.4 software was used to measure the position of the jaw, condyle and its surface bone changes.

**Results:**

Seventeen patients with 20 hypoplastic condyles were included in the study. Among them, 12 joints had disc repositioning by open suturing and 8 by mini-screw anchor. After an average follow-up of 18.1 months, both the TMJ disc and jaw position were stable in the 2 groups except 2 discs moved anteriorly in each group. The overall condylar bone resorption was 8.3% in the open suturing group and 12.5% in the mini-screw anchor group.

**Conclusions:**

Disc repositioning by open suturing can achieve both TMJ and jaw stability for hypoplastic condyles when combined with orthognathic surgery.

## Introduction

Anterior disc displacement (ADD) is a common temporomandibular disorder (TMD). The clinical manifestations are joint pain, clicking, and limited mouth opening. For some cases, conservative treatment such as medications, physiotherapy including low-intensity pulsed ultrasound etc. can achieve good results through masticatory biofeedback [1, 2]. Whereas in some cases, ADD may cause condylar resorption [3]. When ADD happens in juveniles, the growth of the condyle may be affected and cause mandibular asymmetry and/or retrognathia [4]. Studies have showed that ADD may increase jaw instability when combined with orthognathic surgery [5], especially for idiopathic condylar resorption (ICR) patients where the relapse rate has been reported from 83.3% to 100% [6]. To solve this problem, Wolford proposed disc repositioning when combined with orthognathic surgery [7] by Mitek anchors and gained more stable results than when disc repositioning was not performed [8–11]. Later, Yang et. al designed a self-inserted titanium mini-screw anchor (5 mm in length and 2 mm in diameter), with a slot at the end for bolting sutures [12, 13]. But for hypoplastic condyles which has normal morphology and structure but are diminished in size on radiographic examination [14], inserting a metal device (anchor) may interfere with the blood supply of the condyle and cause resorption. In 2001, Yang modified a disc suturing technique under the arthroscope which sutured the disc to the posterior articular capsule [15]. The follow-up results by MRI showed good stability [16]. Later, He used a similar technique as Yang through an open incision to avoid the need for special equipment and for ease of operation [17]. However, the stability of this suturing technique when combined with orthognathic surgery, especially for patients with hypoplastic condyles is unknown.

The purpose of the study was to evaluate TMJ and jaw stability after disc repositioning by open suturing when combined with orthognathic surgery, and to compare it to the Yang’s self-designed mini-screw anchor of disc repositioning for hypoplastic condyles.

## Methods

### Study design

This was a retrospective clinical study which was approved by the Shanghai 9^th^ People’s Hospital Human Research Ethics Committee (SH9H-2018-T88-2). The guidelines of the Declaration of Helsinki were followed in the present study. Patients treated with disc repositioning and simultaneous orthognathic surgery from January 2017 to January 2021 were enrolled in the study. The inclusion criteria were: 1) ADD with hypoplastic condyle (normal condylar morphology but small bone volume) and dentofacial deformity diagnosed by MRI and CT pre-operation; 2) TMJ disc repositioning by either open suturing or mini-screw anchor and concomitant orthognathic surgery (bilateral sagittal split ramus osteotomy, BSSRO ± Le Fort I); 2) operated by one surgeon (Dr. He); 3) MRI and CT data before and within 1 week after operation and at least 6 months follow-up. The exclusion criteria were: 1) ADD with normal condylar morphology and structure; 2) previous TMJ surgery; 3) total joint reconstruction on one side; 4) severely deformed discs which is unsalvageable.

**Fig. 1:**
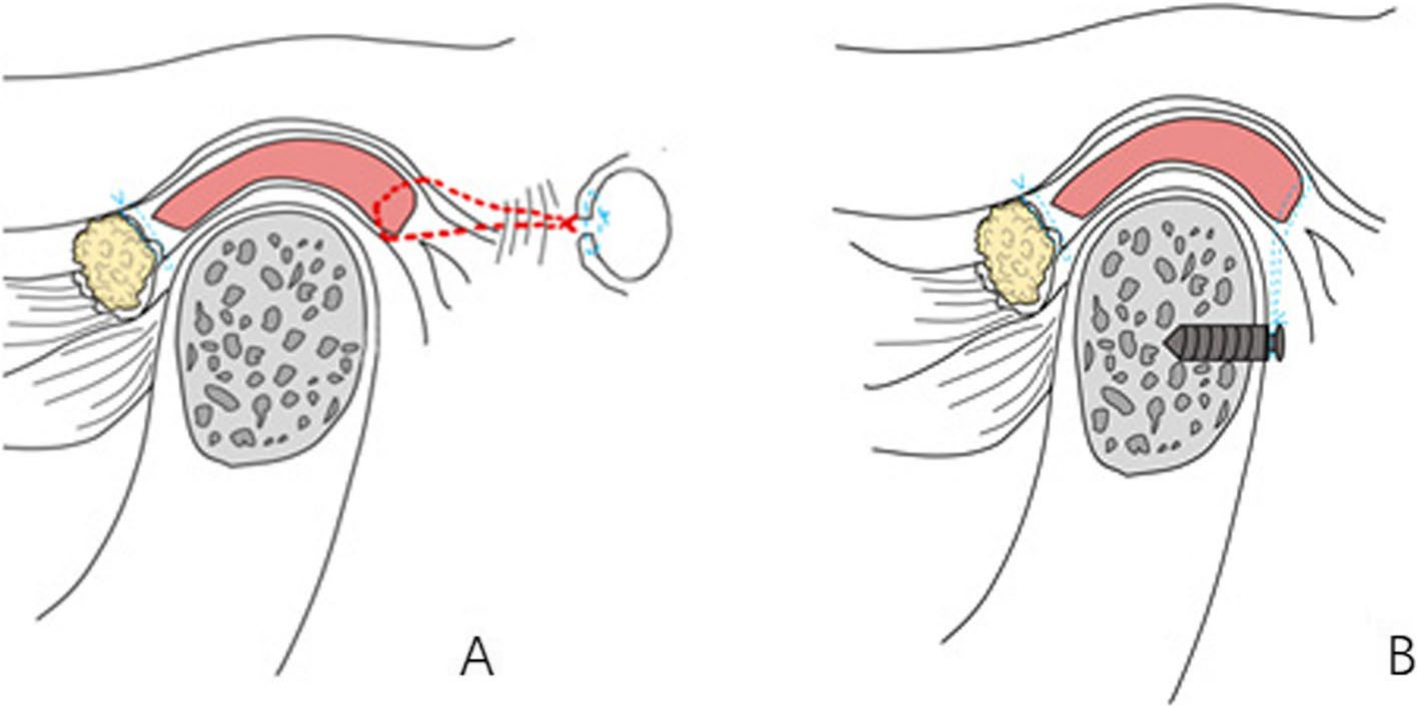
Diagram of disc repositioning methods. **A**, open suturing the disc to the posterior articular capsule; (**B**), mini-screw anchor the disc to the condyle

**Fig. 2:**
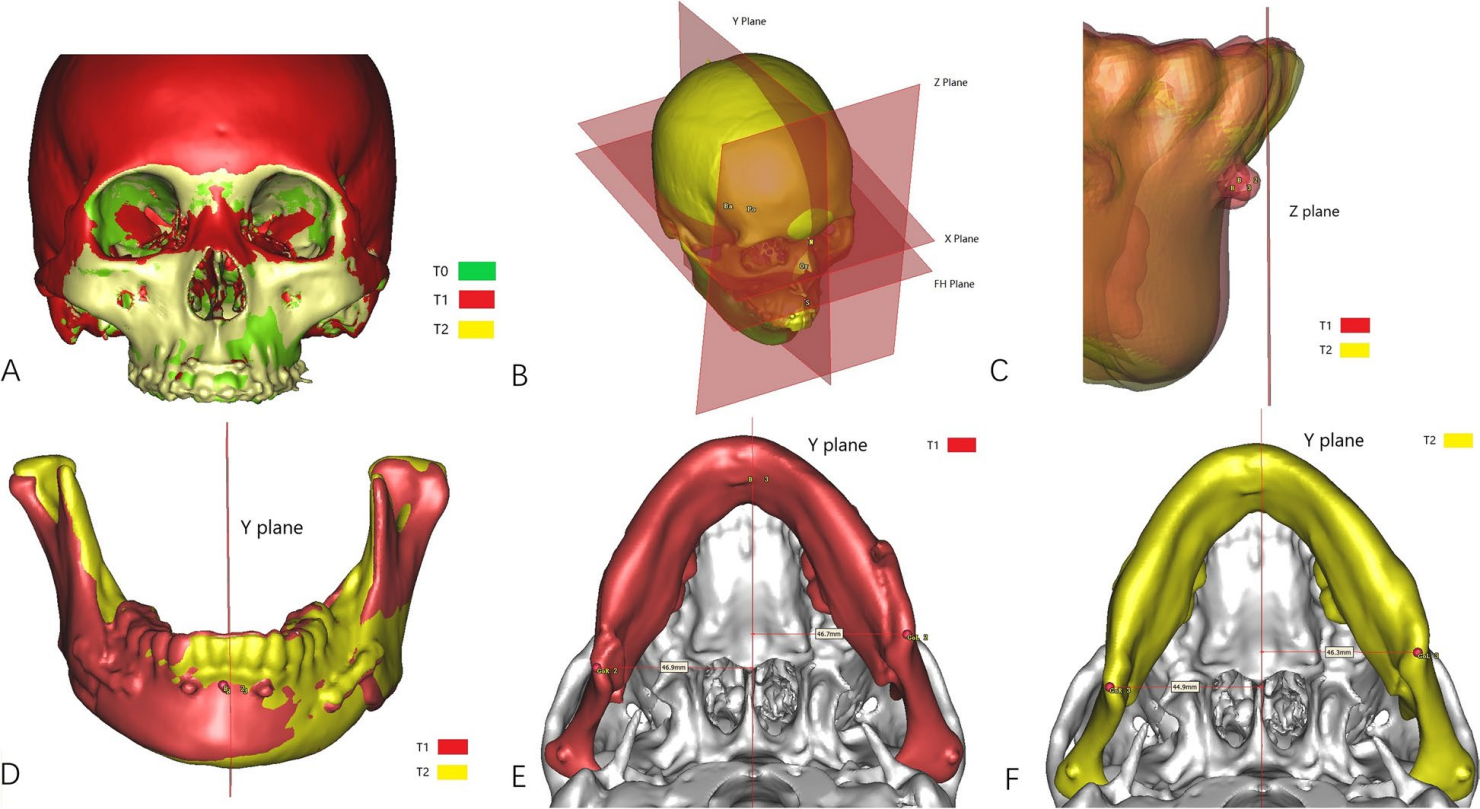
Measurement of the stability. **A**, superimposition of T0, T1 and T2 maxilla models using surface best fit registration; (**B**), coordinate system; (**C**), changes of B-Z between T1 and T2 reflect sagittal stability of mandible; (**D**), changes of B-Y between T1 and T2 reflect coronal stability of mandible; E and F, ΔGo-Y at T1 and T2 reflect mandibular symmetry

Surgical treatment was as follows: 1) disc repositioning by either open suturing to the posterior articular capsule [17] or mini-screw anchor [13] through modified preauricular small incision as we previously described (Fig. 1); 2) BSSRO was performed for all patients. Le Fort I osteotomy was lastly performed when indicated [18].

The TMJ and jaw stability after surgery were evaluated as the following measurements and compared between different disc repositioning methods.

### Variables and measurements

#### TMJ stability

MRI scans were acquired by a 1.5-T imager (Signa, General Electric Medical Systems, Milwaukee, WI) with bilateral 3-inch TMJ surface coil receivers for all patients before and after operation and during follow-ups. Oblique sagittal images with closed- (proton density-weighted imaging, PDWI) and open-mouth (T2-weighted images, T2WI) positions and coronal images (T2WI) of the condyle were acquired for evaluation of disc position and condylar bone status [19]. When the posterior band of the disc was at the 1 to 2 o’clock position of the condyle, it was considered overcorrected and well positioned. When the posterior band of the disc was at the 12 o’clock position of the condyle, it was considered in a normal position. When the posterior band of the disc was anterior to the 12 o’clock position of the condyle and without reduction during mouth opening, it was considered not repositioned or relapsed [20]. The status of condylar bone was defined as bone deposition, no change and resorption.

#### Jaw stability

All patients had CT scans using their maximum intercuspal position (layer thickness, 1 mm; reconstruction layer thickness, 0.625 mm; GE Healthcare, Chicago, IL) and at three time points: preoperative (T0), within 1 week postoperative (T1) and the last follow-up (T2). The position of jaw, condyle and its surface bone changes were measured by ProPlan CMF 1.4 software (Materialise, Leuven, Belgium, Figs. 2A-B). Definitions of three-dimensional (3D) bony landmarks for measurement were shown in Tables 1, 2 and 3.

**Table 1:** Measurement landmarks and definition

Landmarks	Definition
N	Anterior point of frontonasal suture
S	Midpoint of fossa hypophysialis
Ba	Midpoint on anterior margin of foramen occipitale magnum
OrL/OrR, Or	Inferior anterior point on left/right orbit margin and their midpoint
PoL/PoR, Po	Superior point of left/right external acoustic meatus and their midpoint
A	Most posterior point on carve between anterior nasal spine and prosthion
B	Most posterior point on carve between infra dentale and pogonion
GoL/GoR	Most posterior inferior point of left/right ramus
A1	Most anterior point in the midline on alveolar process between upper central incisors
A6L/A6R	Alveolar process below medial buccal cusp of left/right first molar
CoT /CoL/CoM/CoA/CoP	Top/lateral/medial/anterior/posterior point of condyle
CP	Most superior point of coronoid process

**Table 2:** Definition of planes describing maxilla and condylar rotation

Plane	Definition
Maxilla	
Pitch plane	Passing through A, A1 and perpendicular to Y plane
Roll plane	Passing through A6L, A6R and perpendicular to Z plane
Yaw plane	Passing through A6L, A6R and perpendicular to X plane
Condyle	
Pitch plane	Passing through CP, CoT and perpendicular to Y plane
Roll plane	Passing through CoL, CoM and perpendicular to Z plane
Yaw plane	Passing through CoL, CoM and perpendicular to X plane

**Table 3:** Measurement index

Index	Definition
Maxillary stability (°)	
Pitch	Angle between Pitch plane and X plane
Roll	Angle between Roll plane and X plane
Yaw	Angle between Yaw plane and Z plane
Mandibular stability (mm)	
B-Z	Distance between B point and Z plane
B-Y	Distance between B point and Y plane
ΔGo-Y	Difference between the distances of GoL and GoR to Y plane
Condylar rotation (°)	
Pitch	Angle between Co Pitch plane and X plane
Roll	Angle between Co Roll plane and Y plane
Yaw	Angle between Co Yaw plane and Z plane
Condylar movement (°)	
CoT-X	Distance between CoT point and X plane
CoT-Y	Distance between CoT point and Y plane
CoT-Z	Distance between CoT point and Z plane
Condylar remodeling (mm)	
CoT-X	Distance between CoT point and X plane
CoL-Y	Distance between CoL point and Y plane
CoM-Y	Distance between CoM point and Y plane
CoA-Z	Distance between CoA point and Z plane
CoP-Z	Distance between CoP point and Z plane

First, the segmentation function was used to perform 3D reconstruction of the maxillary model with cranial base and mandibular models at T0, T1 and T2 time intervals. As a fixed structure, the cranial base was used for superimposition of T1 and T2 maxillary models to T0 by using surface-best-fit registration as reported by Wan et al. [21]. The position of the maxilla and mandible at different time intervals (T0, T1 and T2) were then compared. The coordinate system was established as: Y plane (sagittal plane): passing through N, S and Ba; FH plane: passing through Or and Po and perpendicular to Y plane; X plane (horizontal plane): passing through N and parallel to FH plane; Z plane (coronal plane): passing through N and perpendicular to X plane and Y plane.

**Fig. 3:**
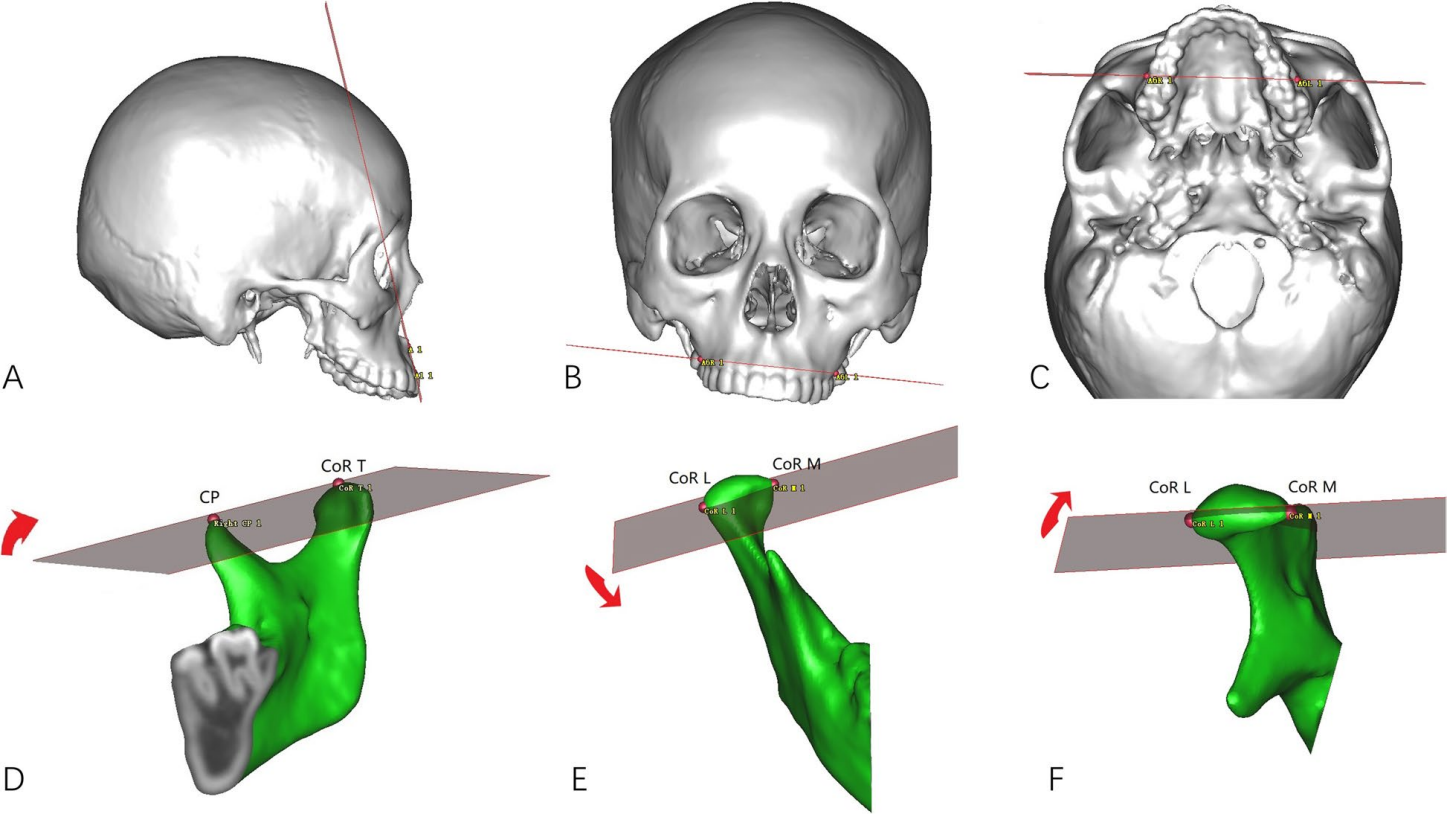
Measurement of the rotation. **A**-**C**, three planes describing maxillary rotation: (**A**), Pitch plane; (**B**), Roll plane, (**C**), Yaw plane. (**D**-**F**), three planes describing condylar rotation: (**D**), Pitch plane; (**E**), Roll plane; (**F**), Yaw plane. Red arrows show the positive direction

Stability of the distal segment of the mandible was measured by the position change of point B in the three-dimensional coordinate system. Stability of the proximal segment of the mandible was measured by condylar rotation and the symmetry of GoR/GoL in the three-dimensional coordinate system. Changes of B-Z between T1 and T2 reflect sagittal stability of the mandible. Changes of B-Y between T1 and T2 reflect coronal stability of the mandible. ΔGo-Y at T1 and T2 reflect mandibular symmetry (Figs. 2C-F). Condylar rotation was described by measuring the changes of Pitch, Roll and Yaw between T1 and T2 mandibular models (Figs. 3A-C). Maxillary stability was measured by the changes of pitch, roll and yaw in the three-dimensional coordinate system between T1 and T2 maxillary models (Figs. 3D-F).

**Fig. 4:**
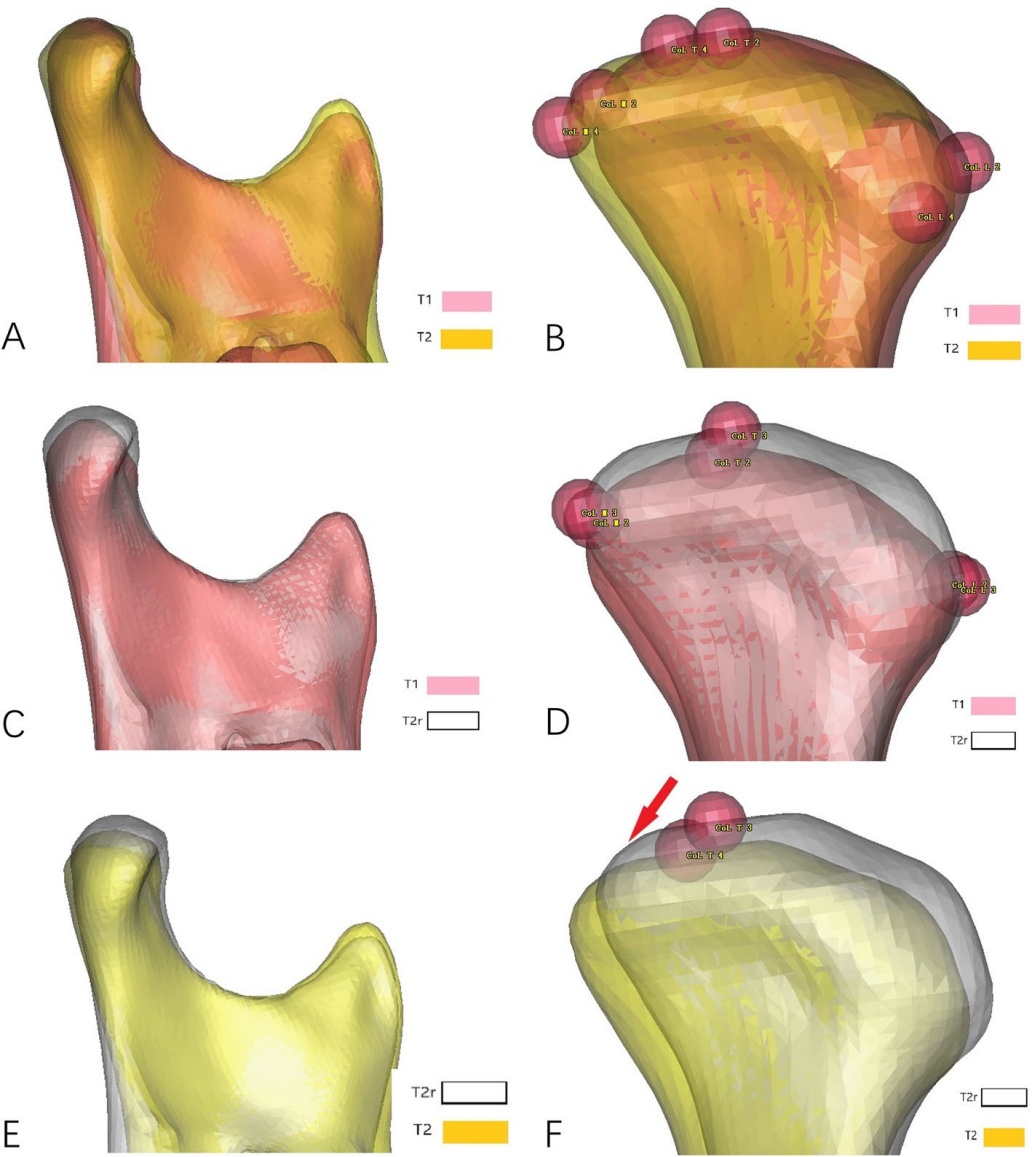
Measurement of the condylar position. **A**-**B**, position changes of correspondent points on condyle between T1 and T2 reflect both condylar movement and remodeling; (**C**-**D**), superimposition of T1 and T2 mandibular models using surface-best-fit registration to measure condylar remodeling; (**E**–**F**), Changes of CoT-X, CoT-Y, CoT-Z between T_2_r and T2 mandible model reflect vertical, lateral and antero-posterior movement of the condyle during follow-up

Condylar position and surface bone remodeling were measured by the position changes of corresponding points between T1 and T2 mandibular models (Figs. 4A-B). In order to distinguish between condylar movement and remodeling, we superimposed T1 and T2 mandibular models using surface-best-fit registration based on the following area of the mandible that are the least affected by bone remodeling after surgery: 1) the posterior area of mandible ramus above the lingua and below the condyle neck; and, 2) the coronoid process [22]. After superimposition, we obtained a registered T2 mandibular model (T_2_r, Figs. 4C-D). Condylar movement was measured by changes of CoT-X, CoT-Y, CoT-Z between T_2_r and T2 mandibular models (Figs. 4E-F) and condylar remodeling was measured by changes of CoT-X, CoL-Y, CoM-Y, CoA-Z, CoP-Z between T_2_r and T2 mandibular models after surgery and during follow-ups (Figs. 3C-D).

### Statistical analysis

Jaw stability, condylar movement and surface bone remodeling in open suturing group and mini-screw anchor group within 1 week after surgery and at the last follow-up were compared by paired *t* test in SPSS 17.0 software (IBM Corp, Armonk, NY). Independent *t* test was used to analyze the differences between open suturing and mini-screw anchor groups. *P* < 0.05 was considered statistically significant.

## Results

**Fig. 5:**
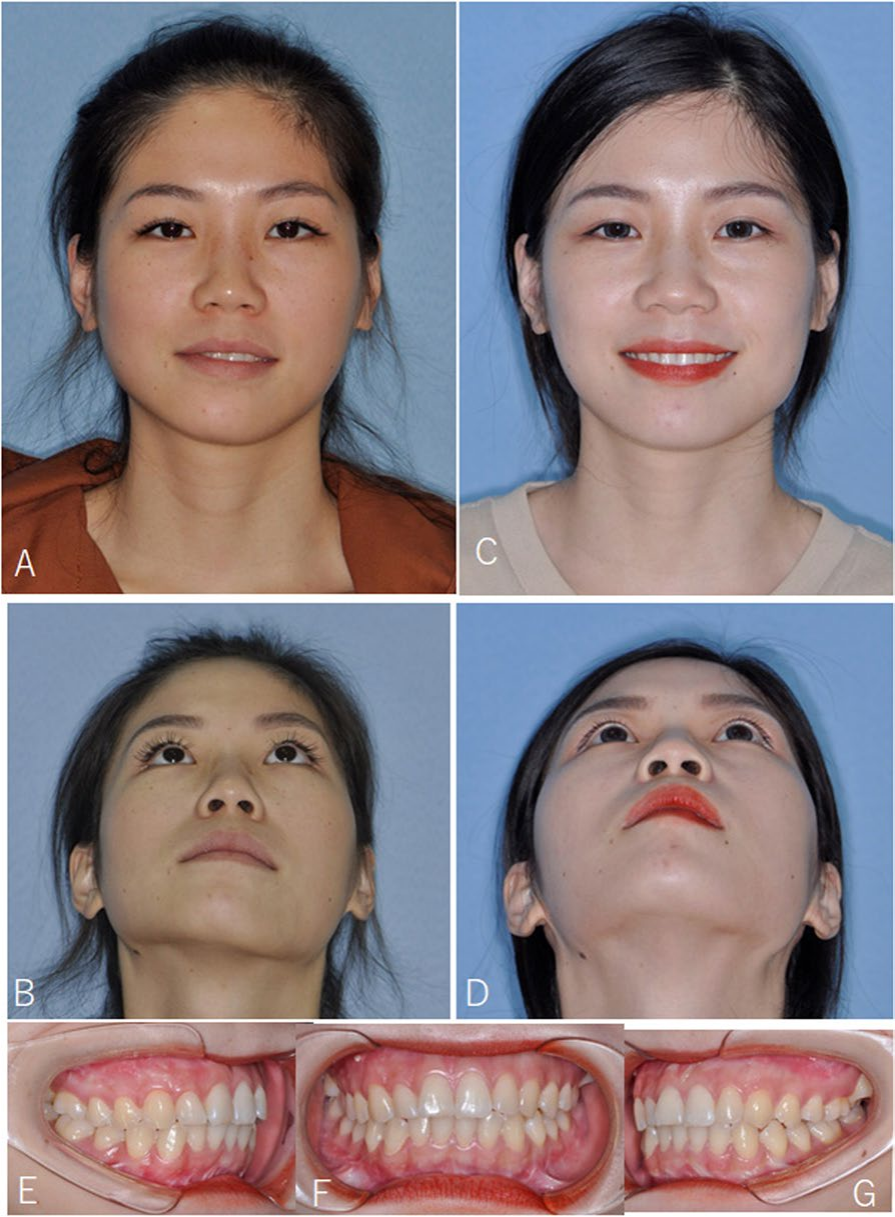
Female, 23 years old with ADD and jaw deviation. **A**, **B**, photos before surgery; (**C**), (**D**), after right TMJ disc repositioning by mini-screw anchor and BSSRO + Le Fort I osteotomy; (**E**–**G**), the occlusion was stable after operation

**Fig. 6:**
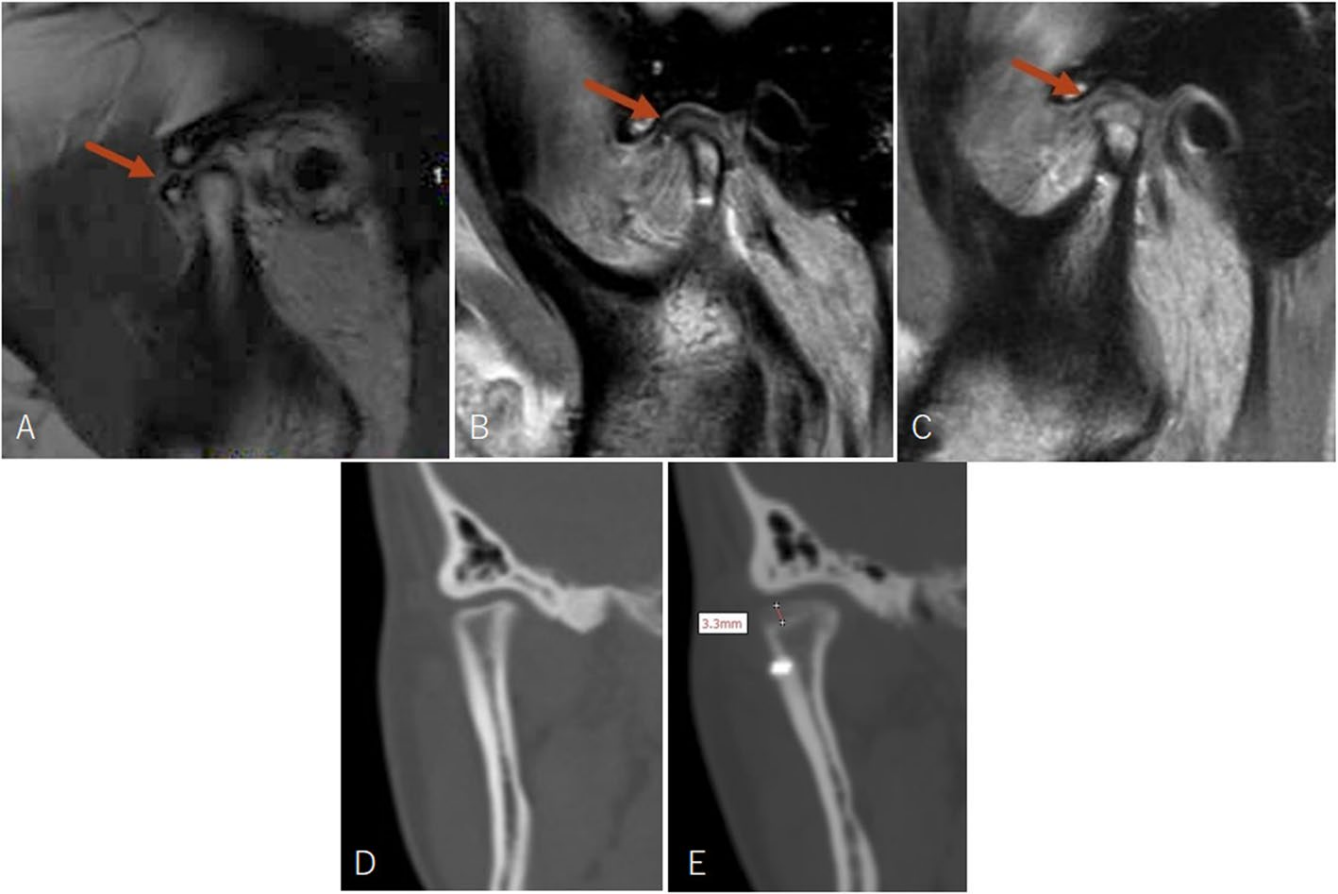
Imaging examinations of patient in Fig. 5. **A**, MRI showed right anterior disc displacement without reduction before surgery; (**B**), the disc was repositioned after surgery; (**C**), the disc was in position at 1.5 years follow-up with significant bone deposition; (**D**), CT showed right condyle had a reduction in volume before surgery; (**E**), significant bone deposition was shown at 1.5 years follow-up. Red arrows indicate TMJ disc

**Fig. 7:**
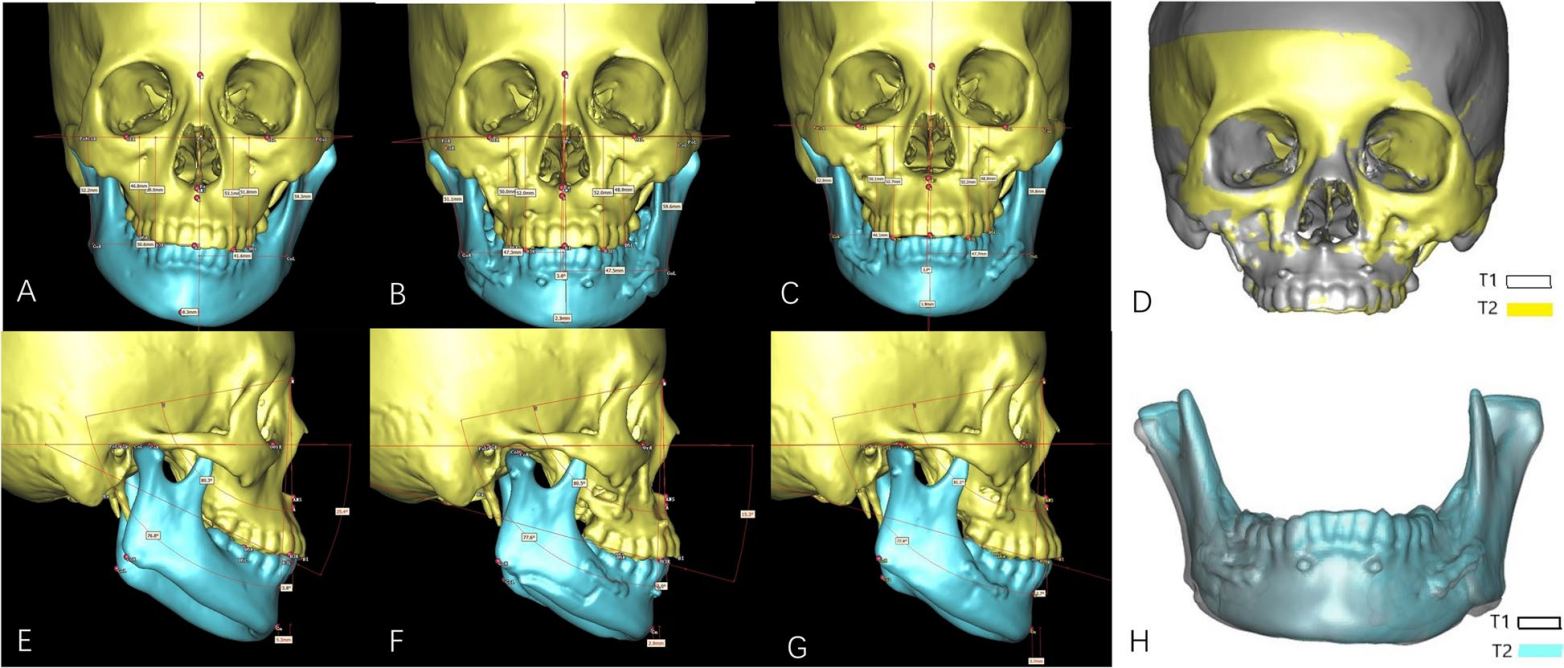
CT measurement of the patient in Fig. 5. **A**, **E**, front view before surgery; (**B**), (**F**), immediate after surgery; (**C**), (**G**), 1.5 years follow-up; (**D**), superimposition of T1 and T2 maxilla models show stable maxilla position. (**H**), superimposition of T1 and T2 mandible models showed bone deposition on the right condyle and stable mandible position

**Fig. 8:**
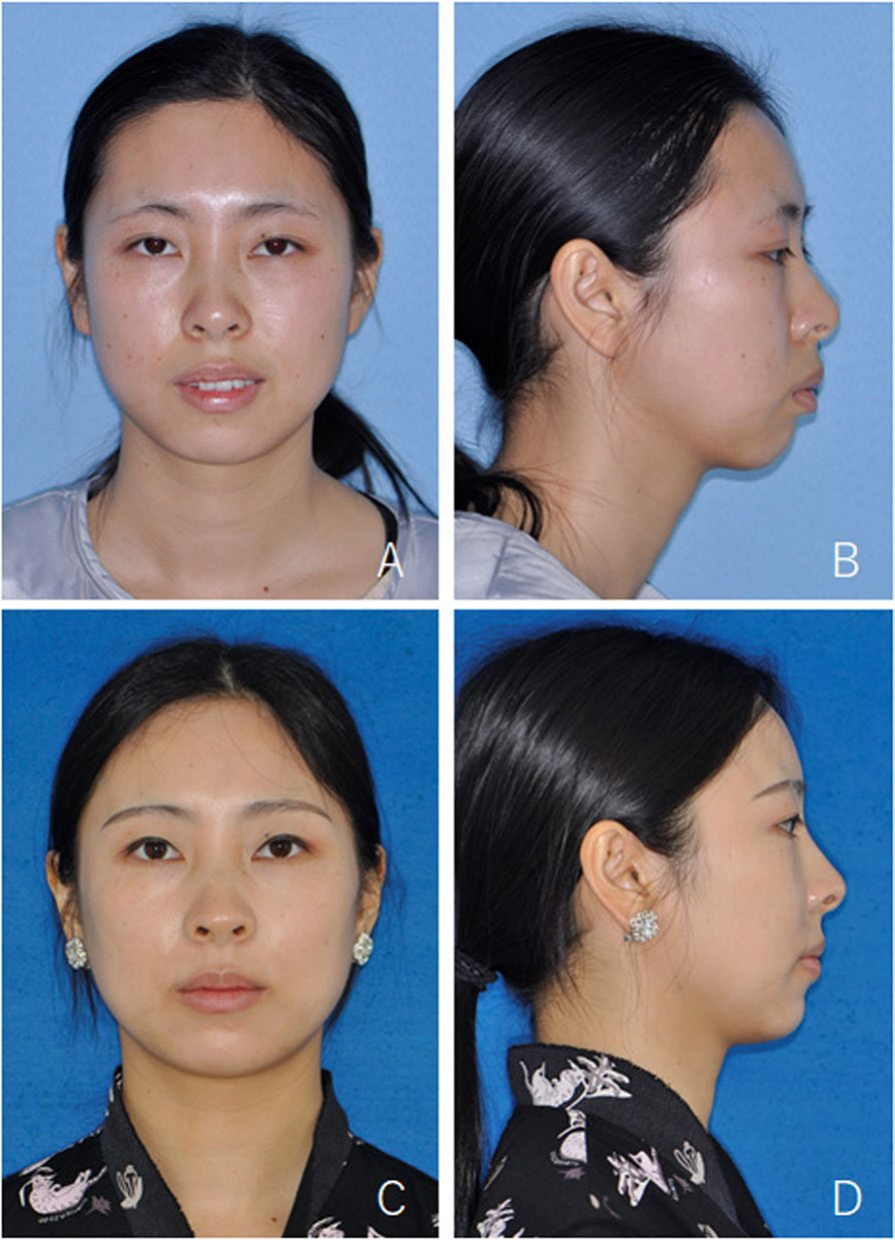
Female, 23 years old with ICR and mandibular retrognathia. **A**, **B**, photos before surgery; (**C**), (**D**), after bilateral TMJ disc repositioning by open suturing and BSSRO + Le Fort I osteotomy

**Fig. 9:**
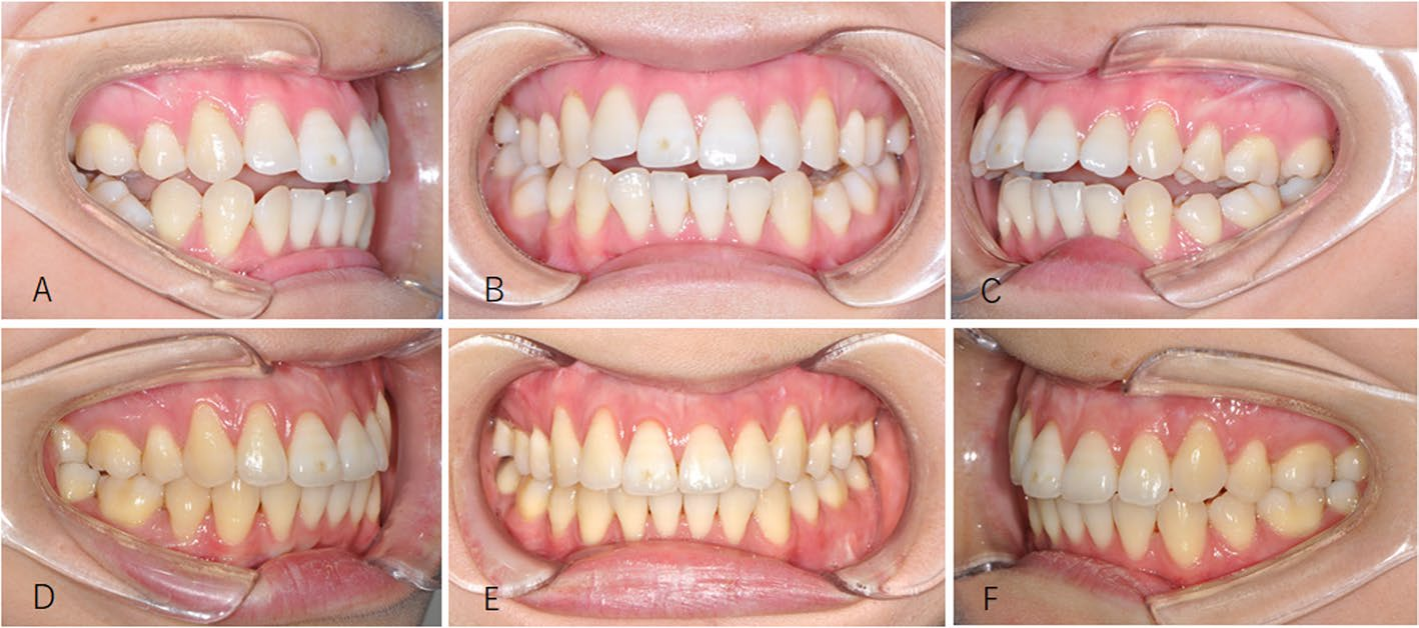
Occlusion of the patient in Fig. 8. **A**-**C**, preoperative occlusion; (**D**)-(**F**), postoperative occlusion after orthodontic treatment

**Fig. 10:**
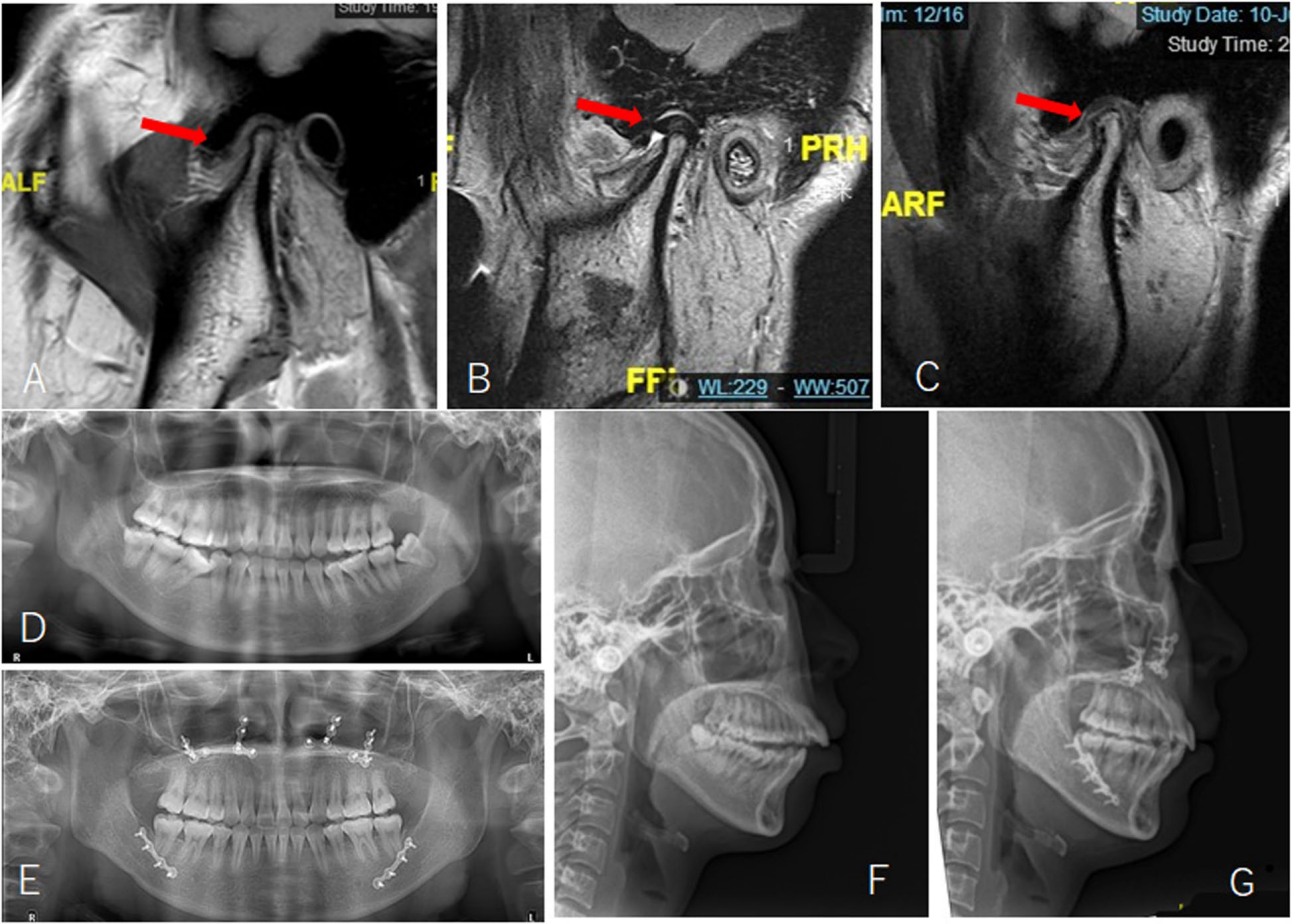
Radiographs of the patient in Fig. 8. **A**, **D**, **F**, preoperative imaging; (**B**), postoperative MRI; (**E**), (**G**), X-rays after orthodontic treatment. Red arrows indicate TMJ disc

Seventeen patients were included in the study. There were 3 males and 14 females ranging in age from 19 to 28 years (mean = 23.1). All patients had TMJ symptoms of joint pain, noises, or mouth opening limitation. Their average follow-up period was 18.7 months (6 to 38 months). Among them, 9 patients with 12 joints had disc repositioned by open suturing, and the other 8 patients with 8 joints by mini-screw anchor. There were 10 patients combined with Le fort I + BSSRO and 7 patients with only BSSRO to correct jaw deformity (Figs. 5, 6, 7, 8, 9 and 10, Table 4). Eight patients were prepared orthodontically to the surgery (4 in suturing group and 4 in mini-screw anchor group). Five patients had “surgery first” and received orthodontic treatment after surgery (2 in suturing group and 3 in mini-screw anchor group). Four patients didn’t receive orthodontic treatment before and after surgery (3 in suturing group and 1 in mini-screw anchor group).

**Table 4:** Patient’s information

	Cases/Joints	Mean Age (year)	Mean FU (month)	BSSRO	LFI + BSSRO	Disc in position (%)
Mini-screw anchor	8/9	22.6	20.3	3	5	8 (88.9%)
Open suturing	9/12	23.4	17.3	4	5	11 (91.7%)
Total	17/21	23.1	18.7	7	10	19 (90.5%)

*FU* follow-up, *BSSRO* bilateral sagittal split ramus osteotomy, *LFI* Le Fort I

The first outcome of this study was the difference in TMJ stability using suturing compared to mini-screw fixation. MRI showed that all the 21 anteriorly displaced discs were repositioned after operation. During follow-ups, 19 discs were in good position but 2 moved anteriorly, including 1 in open suturing group and 1 in mini-screw anchor group. In the open suturing group, 8 condyles had bone deposition (66.7%), 3 had no change (25%) and 1 had a slight bone resorption (8.3%). In the mini-screw anchor group, 3 condyles had bone deposition (37.5%), 4 had no change (50%) and 1 had a slight bone resorption (12.5%). Condylar bone was more stable in the open suturing group than mini-screw anchor group (91.7% vs. 87.5%, Table5). Two condyles with slight bone resorption had anteriorly disc relapse.

**Table 5:** Status of bone after disc repositioning by MRI

Condylar bone status	Open suturing	%	Mean age	Mini-screw	%	Mean age
Bone deposition	8	66.7%	23.4	3	37.5%	22.3
No change	3	25%	23.3	4	50%	21.5
Bone resorption	1	8.3%	24	1	12.5%	28
Total	12	100%	23.4	8	100%	22.6

The second outcome was jaw stability after disc repositioning by open suturing or mini-screw anchor for hypoplastic condyles when combined with orthognathic surgery. CT measurement showed that there were no significant jaw position changes in both open suturing and mini-screw anchor groups during follow-up (*p* > 0.05, Table 6). Although immediately after surgery, condyles after disc repositioning moved significantly downward (average of 1.57 mm, *p* = 0.000) and laterally (average of 1.12 mm, *p* = 0.007), and at the last follow up, they moved upward (average of 1.24 mm, *p* = 0.000), medially (average of 0.73 mm, *p* = 0.009) and posteriorly (average of 1.16 mm, *p* = 0.001, Table 7), jaw stability was not affected. At the last follow-up, condyles after disc repositioning showed significant bone deposition on the medial surface (*p* = 0.043, Figs. 6C, E 10C and Table 8). There was no significant bone resorption in the two groups (*p* > 0.05, Table 9).

**Table 6:** Comparison of jaw stability between mini-screw anchor group and open suturing group

Index	ΔT1-T2		p value
	Mini-screw anchor	Open suturing	
Mandibular stability (mm)			
B-Z	1.68 ± 0.98	1.59 ± 1.80	0.906
B-Y	1.38 ± 0.86	0.99 ± 0.87	0.373
ΔGo-Y	0.98 ± 2.24	0.02 ± 1.46	0.304
Rotation of the operated condyle (°)			
Pitch	2.34 ± 1.57	1.31 ± 1.60	0.165
Roll	1.06 ± 0.97	1.21 ± 0.77	0.698
Yaw	1.10 ± 1.10	1.35 ± 1.18	0.639
Rotation of the non-operated condyle (°)			
Pitch	1.86 ± 1.83	1.46 ± 1.58	0.704
Roll	1.19 ± 1.16	0.78 ± 0.57	0.491
Yaw	0.70 ± 1.00	1.32 ± 1.18	0.349
Maxillary stability (°)			
Pitch	3.06 ± 3.43	1.98 ± 1.92	0.556
Roll	1.34 ± 0.72	0.52 ± 0.36	0.053
Yaw	1.32 ± 0.91	0.82 ± 1.39	0.521

*T1* immediate after operation, *T2* at the last follow-up. *p < 0.05

**Table 7:** Condylar movement after operation and at the last follow-up

Index	T0	T1	ΔT0-T1		T2	ΔT1-T2	
	Mean	Mean	Mean ± SD	p value	Mean	Mean ± SD	p value
Movement of non-operated condyles							
CoT-X(upward-downward)	27.25	27.42	-0.17 ± 0.66	0.374	26.71	0.71 ± 1.15	0.047*
CoT-Y (lateral-medial)	54.35	54.45	0.11 ± 1.62	0.815	53.90	-0.55 ± 0.56	0.004**
CoT-Z (anterior–posterior)	72.01	72.35	-0.35 ± 1.29	0.352	72.37	-0.02 ± 1.18	0.945
Movement of operated condyles							
CoT-X(upward-downward)	29.40	30.98	-1.57 ± 1.24	0.000**	29.74	1.24 ± 1.20	0.000**
CoT-Y (lateral-medial)	53.79	54.60	1.12 ± 1.74	0.007**	53.87	-0.73 ± 1.16	0.009**
CoT-Z (anterior–posterior)	71.75	71.42	0.32 ± 1.49	0.331	72.58	-1.16 ± 1.44	0.001**

*T1* immediate after operation, *T2* at the last follow-up. *p < 0.05, **p < 0.01

**Table 8:** Bone remodeling of affected condyles (mm)

Index	T1	T2	ΔT1-T2	p value
	Mean	Mean	Mean ± SD	
CoT-X(superior)	30.88	30.66	0.22 ± 0.84	0.245
CoA-Z (anterior)	67.65	67.36	0.29 ± 0.75	0.097
CoP-Z(posterior)	76.41	76.23	-0.18 ± 0.75	0.284
CoL-Y(lateral)	61.96	61.82	-0.13 ± 0.49	0.225
CoM-Y(medial)	45.35	45.00	0.35 ± 0.75	0.043*

*T1* immediate after operation, *T2* at the last follow-up. *p < 0.05

**Table 9:** Comparison of bone remodeling of affected condyles between mini-screw anchor group and open suturing group (mm)

Index	ΔT1-T2		p value
	Mini-screw anchor	Open suturing	
CoT-X(superior)	0.18 ± 1.00	0.25 ± 0.74	0.851
CoA-Z (anterior)	0.14 ± 0.46	0.39 ± 0.92	0.470
CoP-Z(posterior)	-0.03 ± 0.57	-0.29 ± 0.87	0.451
CoL-Y(lateral)	-0.22 ± 0.54	-0.07 ± 0.46	0.484
CoM-Y(medial)	0.20 ± 0.95	0.47 ± 0.58	0.433

*T1* immediate after operation, *T2* at the last follow-up

## Discussion

The TMD prevalence is increasing, especially during pandemic COVID 19, therefore new treatment methods are especially needed [23]. As the most common type of TMD, presurgical ADD is an important factor for the relapse of orthognathic surgery [24, 25]. Disc repositioning for ADD when combined with orthognathic surgery by Mitek anchors have been reported with stable results [7–11]. In this study, we tried a new method of disc repositioning by open suturing for hypoplastic condyles when combined with orthognathic surgery. Instead of inserting a metal anchor to the condyle, we used non-absorbable suture to fix the disc into the posterior capsule. The advantage of this method is no disturbance of the blood supply to the condyle during dissection. The disadvantage is the stability of fixation the disc to soft tissue instead of bone, especially when the jaw was split and moved during orthognathic surgery. We used MRI and CT measurements to evaluate TMJ and jaw stability in this study. The results showed that both techniques (open suturing and mini-screw anchor) for disc repositioning on hypoplastic condyles can acquire stable TMJ and jaw position when combined with orthognathic surgery.

TMJ stability was evaluated by MRI which is a golden standard for disc position and bone status. After orthognathic surgery, each group had 1 disc relapsed anteriorly and slight bone resorption. Compared with mini-screw anchor, open suturing had more condylar bone deposition (66.7% vs. 37.5%), and less bone resorption (8.3% vs. 11.1%) with an overall more condylar bone stability (91.7% vs. 87.5%). Gomes et al. found after disc repositioning by Mitek anchors when combined with orthognathic surgery, the overall condylar volume tended to reduce and over 30% of the cases showed more than 1.5 mm of bone resorption on at least one surface of the condyle [8]. But he did not describe the jaw stability. In our study, we also found 12.5% of the hypoplastic condyles with ADD had slight bone resorption about 1 mm after disc repositioning by Yang’s self-designed mini-screw anchor, but the jaw stability was not affected. So far, there is no report on the correlation between the degree of condylar resorption and jaw stability with orthognathic surgery. How much condylar resorption which affects jaw stability needs further study.

After TMJ disc repositioning, the condyle moved downward and laterally, but during follow-ups, it moved upward, medially and posteriorly. This is because of the reduction of postsurgical swelling and disc reshaping developed as Chen and Gomes reported [26, 27]. Although the condyle moved a little bit, the patient’s jaw position and occlusion were stable within the range of compensation.

Small sample size and the predominance of women in the group were the limitations of this study. Since it was a preliminary study on the new disc repositioning method by open suturing when combined with orthognathic surgery, we only tried a small number of patients to see if it was stable before large number of patients performed. According to the pilot data, a prospective study will be designed in the future including adequate number of patients for further analysis.

## Conclusions

Disc repositioning by open suturing can acquire stable TMJ and jaw position when combined with orthognathic surgery. The condyles had good adaptative changes after surgery during mandibular function.

## Data Availability

The data collected and analyzed in the current study are not publicly available due to ethical restrictions, but are available from the corresponding author upon reasonable request.

## Abbreviations

ADD: Anterior disc displacement
TMJ: Temporomandibular joint
ICR: Idiopathic condylar resorption
BSSRO: Bilateral sagittal split ramus osteotomy
